# Is a Combination of Six Clinical Tests Useful as a Measure to Predict Short-Term Prognosis in Terminal Cancer Patients? A Prospective Observational Study in a Japanese Palliative Care Unit

**DOI:** 10.1089/pmr.2024.0026

**Published:** 2024-10-09

**Authors:** Kazuyuki Niki, Yoshiaki Okamoto, Maki Yasui, Takahito Omae, Makie Kohno, Yoshinobu Matsuda

**Affiliations:** ^1^ Department of Clinical Pharmacy Research and Education, Osaka University Graduate School of Pharmaceutical Sciences, Osaka, Japan.; ^2^ Department of Pharmacy, Ashiya Municipal Hospital, Hyogo, Japan.; ^3^ Omae Homecare Clinic, Amagasaki, Japan.; ^4^ Department of Palliative Care, Ashiya Municipal Hospital, Hyogo, Japan.

**Keywords:** laboratory test value, objective prognostic prediction, palliative care unit, patient with terminal cancer

## Abstract

**Background:**

To address the need for short-term prognostic methods using objective measures, we developed a method to predict a 2- or 3-week prognosis using only six clinical tests (known as the WPCBAL score). However, the method has not yet been directly compared with globally accepted prognostic methods.

**Objectives:**

This study aimed to clarify the usefulness of the WPCBAL score by comparing it with other prediction methods.

**Setting/Subjects:**

A prospective observational study was conducted with patients admitted to the palliative care unit of a Municipal Hospital in Japan between November 2017 and May 2021.

**Measurements:**

The primary endpoint was each prediction method’s accuracy—the WPCBAL score, Glasgow Prognostic Score (GPS), Palliative Prognostic Index (PPI), Palliative Prognostic Score (PaP), Delirium-Palliative Prognostic Score (D-PaP), and Prognosis in Palliative Care Study predictor models (PiPS-A, PiPS-B)—in predicting a prognosis at 2 or 3 weeks. The secondary endpoints were sensitivity, specificity, positive and negative predictive values, area under the receiver operating characteristic curve, and each prediction method’s feasibility rate.

**Results:**

In total, 181 patients were included in this study. For the 3-week prognosis, the PaP had the highest accuracy (0.746), followed by the D-PaP (0.735), WPCBAL (0.696), PPI (0.652), and GPS (0.575). For the 2-week prognosis, the PiPS-B had the highest accuracy (0.702), followed by the WPCBAL (0.696) and PiPS-A (0.641).

**Conclusions:**

The WPCBAL score’s accuracy in predicting a 2- or 3-week prognosis was comparable to that of commonly used prognostic methods, thus suggesting its usefulness as a short-term prognostic method.

## Introduction

In palliative care, prognostic information is important for patients with terminal cancer and their families and caregivers. It is especially necessary for patients and their families to optimize their remaining time^1^ and prepare for death,^2^ and for physicians to appropriately determine when to discontinue active treatment and transfer the patient to a palliative care unit.^3–5^ Therefore, various methods have been developed to predict the prognosis of patients with terminal cancer, such as the Palliative Prognostic Score (PaP),^6^ Delirium-Palliative Prognostic Score (D-PaP),^7^ Palliative Prognostic Index (PPI),^8^ Glasgow Prognostic Score (GPS),^9^ and Prognosis in Palliative Care Study predictor models (PiPS-A, PiPS-B).^10^

Although several prognostic methods have been developed, the lack of a simple method to determine a short-term prognosis and the focus of most methods on subjective evaluation items means that prediction results are dependent on physician experience.^11,12^ Therefore, there is a need for objective prognostic methods. Hui et al. recently reported that prognosis within 30 days is more accurate when subjective assessments are excluded.^13^ In 2015, the GPS^9^ was reported to be more sensitive than the PPI^8^ for predicting a prognosis at 3 weeks.^14^ In 2016, the Objective Palliative Prognostic Score was reported as a potential method for predicting a 1-week prognosis,^15^ but evidence for its usefulness was insufficient. The objective method by Hamano et al.^16^ to predict a 2-week prognosis is the only currently available method. Predicting a 2-week prognosis is particularly important for determining the course of treatment. For example, at least 2 weeks are needed for antidepressants to take effect, and side effects are more of a concern than efficacy when the prognosis is within 2 weeks. Midazolam, which is commonly used for sedation at the end of life, may be tolerated if used for more than 2 weeks.^17^ A previous study focused on nonspecific life-threatening complications immediately before death and the deterioration of the general condition regardless of cancer type.^18^ The study hypothesized that fluctuations in laboratory values may occur as death approaches. A method to objectively predict short-term prognosis was developed using only the combined score of six laboratory tests (white blood cells [WBC], platelets [PLT], C-reactive protein [CRP], blood urea nitrogen [BUN], aspartate transaminase [AST], and lactate dehydrogenase [LDH]), known as the WPCBAL score. This method can be used in routine clinical practice and predict a prognosis at 2 weeks with high accuracy.^19^ The first study of WPCBAL scores examined laboratory test results obtained within 90 days before a person’s death, with weekly monitoring. The laboratory tests included 15 parameters, including WBC, PLT, CRP, BUN, AST, and LDH. Changes in these values were analyzed using differential calculus to detect change points. The laboratory test values that included the top 70% of patients from 1 to 2 weeks before death were set as reference values for WBC, BUN, AST, LDH, and CRP. Similarly, the laboratory test values that included the bottom 70% of patients from 1 to 2 weeks before death were set as reference values for PLT.

Despite blood tests being essential for obtaining laboratory values, blood sampling is generally difficult in home or hospice settings.^20,21^ A previous study used data from 2008 to narrow down useful laboratory items for prognosis prediction in acute care wards where blood tests are routinely performed.^19^ However, although the patients in this study had cancer, they were not end-of-life patients in a palliative care unit because many were undergoing chemotherapy. A retrospective cohort study conducted among patients admitted to a palliative care unit in 2016 suggested that the WPCBAL score could be a new method for predicting prognosis at 2 or 3 weeks based solely on objective measures.^22^ However, because this was a retrospective cohort study, a direct comparison with globally accepted prognostic methods could not be performed. To address this gap, the current prospective observational study compared the WPCBAL score with other prediction methods and examined its usefulness.

## Methods

### Eligible patients

We included patients with cancer who were admitted to the Department of Palliative Care at Ashiya Municipal Hospital from November 1, 2017 to May 31, 2021 (*n* = 687) and patients whose prognostic evaluation was performed by a physician within 3 days of admission (*n* = 584). Among them, we excluded patients whose laboratory test (WBC, PLT, BUN, AST, LDH, CRP, albumin [ALB], alanine aminotransferase [ALT], alkaline phosphatase [ALP], neutrophil count, lymphocyte count, and lymphocyte percentage) and heart rate data were missing and those whose blood test dates were more than 4 days from the date of admission. Blood samples were drawn when deemed necessary by the physician. Generally, the following patients are admitted to palliative care units in Japan: patients evaluated as difficult to treat or who do not wish to be treated for cancer or acquired immune deficiency syndrome (AIDS); patients with mental or physical symptoms associated with cancer or AIDS; patients with difficulty recuperating at home or elsewhere; patients judged by a physician to require hospitalization; and patients or their relatives who wish for them to be admitted to a palliative care unit.

### Study design

This was a prospective observational study.

### Implementation of prognostic prediction

Within the scope of daily practice, physicians in the palliative care unit predicted the prognosis of hospitalized patients using the PPI, PaP, D-PaP, PiPS-A, PiPS-B, GPS, and WPCBAL scores. Two physicians made the predictions—one had 40 years’ experience as a physician with involvement in palliative care for 18 years, the other had been a physician for 14 years with 10 years’ involvement in palliative care. We obtained the following data from medical records: age, sex, body mass index (BMI), Eastern Cooperative Oncology Group Performance Status (ECOG PS), primary cancer site, prior chemotherapy, prior radiation therapy, number of days of survival since admission to the palliative care unit, laboratory values (WBC, PLT, BUN, AST, LDH, CRP, ALB, ALT, ALP, neutrophil count, lymphocyte count, and lymphocyte percentage), and heart rate. The difference between the dates of admission and death of patients who were alive for more than 3 weeks but whose date of death was unknown was considered as “prognosis >3 weeks” and included in the analysis.

### Feasibility rate for each prediction method

We calculated the percentage of patients whose prognosis could be predicted using each prediction method. Cases that were not predictable were defined as those with missing data.

### Calculation of accuracy, sensitivity, specificity, positive and negative predictive values, and area under receiver operating characteristic curve

The accuracy, sensitivity, specificity, positive predictive value (PPV), and negative predictive value (NPV) of each prediction method were calculated by creating 2 × 2 contingency tables for PPI, PaP, D-PaP, GPS, and WPCBAL scores to determine whether death occurred within 3 weeks of the prediction time; similar tables were also created for PiPS-A, PiPS-B, and WPCBAL scores to determine whether death occurred within 2 weeks of the prediction time. The cutoff values for each prediction method followed those reported^6–8,10,14^ when each prediction method was validated. Factors comprising the WPCBAL score, GPS, and reference values are listed in Table 1. A previous study reported that the cutoff values for the WPCBAL score and GPS are 4 and 5 points, respectively, for the 3-week and 2-week prognoses.^22^ The WPCBAL score and GPS are predictive methods that use a scoring system in which one point is given if the criterion value for the test item is met. The cutoff value of the GPS score at 3 weeks was reported to be two points.^14^ In calculating the area under the receiver operating characteristic (AUROC) curve for the 3-week prognosis, the objective variable was the presence or absence of death within 3 weeks, and the scores of each prediction method (PPI, PaP, D-PaP, GPS, and WPCBAL) were used as explanatory variables. To predict the 2-week prognosis, the objective variable was the presence or absence of death within 2 weeks, and the scores of each prediction method (PiPS-A, PiPS-B, and WPCBAL) were used as explanatory variables.

**Table 1.** Reference Values of Predictors for the WPCBAL Score and Glasgow Prognostic Score

Predictive method	Predictor and reference value
WPCBAL score	WBC ≥4.87 × 10^3^/μL
	PLT ≤225 × 10^3^/μL
	CRP ≥2.75 mg/dL
	BUN ≥14 mg/dL
	AST ≥30U/L
	LDH ≥264U/L
GPS	ALB <3.5 g/dL
	CRP >1.0 mg/dL

ALB, serum albumin; AST, aspartate aminotransferase; BUN, blood urea nitrogen; CRP, C-reactive protein; GPS, Glasgow prognostic score; LDH, lactate dehydrogenase; PLT, platelet; WBC, white blood cell.

### Calculation of sample size

The sample size was based on the necessary population proportion of 50% and was calculated to maintain the width of the 95% confidence interval within 15%, as in a previous study.^23^ This resulted in a required sample size of 171 individuals. Therefore, the target number of cases for this study was set at 180.

### Primary and secondary endpoints

The primary endpoint was the accuracy of each method in predicting a prognosis at 2 or 3 weeks. The secondary endpoints were sensitivity, specificity, PPV, NPV, AUROC, and the feasibility of each prediction method.

### Statistical analysis

Statistical significance was defined as *p* < 0.05. The Bell Curve for Excel (Social Survey Research Information Co., Ltd., Tokyo, Japan) was used for the analysis.

## Results

### Patient characteristics

In total, 584 patients with cancer were admitted to the Department of Palliative Care at Ashiya Municipal Hospital from November 1, 2017 to May 31, 2021, and prognostic predictions were performed by physicians. We included 181 patients in the study after excluding those with missing laboratory values (305 patients), missing subjective physician evaluation items (32 patients), or blood test data dated more than 4 days from the date of admission (216 patients). As this study also examined the feasibility rate of implementing the WPCBAL score, the total number of patients who fit each exclusion criterion is shown above, but they included duplicates among the exclusion criteria. Table 2 summarizes the patient characteristics. The median patient age was 79 years (interquartile range [IQR]: 71–87), and 162 patients (89.5%) had ECOG PS ≥3. The median survival time was 19 days (IQR: 9–42 days). In total, 67 patients (37.0%) died within 2 weeks and 99 patients (54.7%) died within 3 weeks of enrollment.

**Table 2.** Patients’ Demographic Characteristics

Characteristics	Value
*N*	181
Age, median (IQR)	79.0 (71–87)
Male, *n* (%)	91 (50.3)
BMI, median (IQR), (*n* = 98)	19.5 (17.1–21.6)
ECOG PS, *n* (%)	
3≥	162 (89.5)
2≤	19 (10.5)
Primary cancer site, *n* (%)	
Lung	32 (17.7)
Colon and rectum	30 (16.6)
Stomach	18 (9.9)
Pancreas	18 (9.9)
Breast	17 (9.4)
Blood	9 (5.0)
Esophagus	7 (3.9)
Liver	6 (3.3)
Kidney	6 (3.3)
Uterus	5 (2.8)
Prostate	4 (2.2)
Urinary bladder	4 (2.2)
Ovary	4 (2.2)
Head and neck	4 (2.2)
Bile duct	3 (1.7)
Others	10 (5.5)
History of chemotherapy, *n* (%)	103 (56.9)
History of radiotherapy, *n* (%)	43 (23.8)
Duration between hospitalization and death, days, median (IQR), (*n* = 177)	19 (9–42)

BMI, body mass index; ECOG PS, Eastern Cooperative Oncology Group Performance Status; IQR, interquartile range.

### Comparison of the predictive ability of each method

**FIG. 1.**
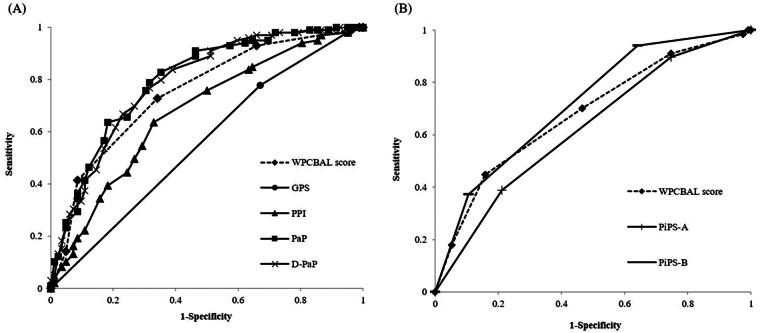
Receiver operating characteristic curve analysis. The WPCBAL score, GPS, PPI, PaP, D-PaP, PiPS-A, and PiPS-B were defined as independent variables, and death within 3 weeks **(A)** or 2 weeks **(B)** after admission was defined as the dependent variable.

Table 3 shows the accuracy, sensitivity, specificity, PPV, NPV, and AUROC of each prediction method. The ROC curves for each method are shown in Figure 1. In the 3-week prognosis projection, the PaP had the highest accuracy, followed by the D-PaP, WPCBAL score, PPI, and GPS. The D-PaP exhibited the highest sensitivity, followed by the PaP, GPS, WPCBAL score, and PPI. Specificity was highest for the PPI, followed by the WPCBAL score, PaP, D-PaP, and GPS. Furthermore, the PaP had the highest PPV, followed by the D-PaP, WPCBAL score, PPI, and GPS. The D-PaP had the highest NPV, followed by the PaP, WPCBAL score, PPI, and GPS. The AUROC was highest for the PaP, followed by the D-PaP, WPCBAL score, PPI, and GPS; however, the PaP, D-PaP, and WPCBAL scores were all above 0.7, with *p* < 0.001. The AUROC of the WPCBAL score was also significantly higher than that of the GPS (*p* < 0.001) but not significantly different from that of the other prediction methods.

**Table 3.** Accuracy, Sensitivity, Specificity, Positive and Negative Predictive Values, and Area under the Receiver Operating Characteristic Curve of Each Prognostic Predictive Method: (a) 3-Week Prediction, (b) 2-Week Prediction

Methods	Category	Cutoff	Accuracy	Sensitivity	Specificity	PPV	NPV	AUROC (95% CI)	AUROC *p* value	AUROC *p* value (vs. WPCBAL)
(a)										
WPCBAL score	6	≧4/≦3	0.696	0.727	0.659	0.720	0.667	0.7514 (0.6822–0.8206)	<0.001	—
GPS	2	2/≦1	0.575	0.778	0.329	0.583	0.551	0.5559 (0.4900–0.6218)	0.0964	<0.001
PPI	5	≧6/<6	0.652	0.636	0.671	0.700	0.604	0.6743 (0.5955–0.7532)	<0.001	0.1467
PaP	6	≧9/<9	0.746	0.828	0.646	0.739	0.757	0.7965 (0.7307–0.8623)	<0.001	0.3217
D-PaP	7	≧9/<9	0.735	0.838	0.610	0.722	0.758	0.7882 (0.7212–0.8553)	<0.001	0.4141
(b)										
WPCBAL score	6	≧5/≦4	0.696	0.448	0.842	0.625	0.722	0.6827 (0.6035–0.7619)	<0.001	—
PiPS-A	13	days/weeks, months	0.641	0.388	0.789	0.520	0.687	0.6254 (0.5499–0.7009)	0.0011	0.3055
PiPS-B	19	days/weeks, months	0.702	0.373	0.895	0.676	0.708	0.7200 (0.6546–0.7854)	<0.001	0.3868

AUROC, area under the receiver operating characteristic curve; CI, confidence interval; D-PaP, delirium-palliative prognostic score; GPS, Glasgow prognostic score; NPV, negative predictive value; PaP, palliative prognosis score; PiPS, prognosis in palliative care study predictor models; PPI, Palliative Prognostic Index; PPV, positive predictive value.

In the 2-week prognosis projection, the PiPS-B had the highest accuracy, followed by the WPCBAL score and PiPS-A. The WPCBAL score had the highest sensitivity, followed by the PiPS-A and PiPS-B. The PiPS-B had the highest specificity, followed by the WPCBAL score and PiPS-A. The PPV was the highest for PiPS-B, followed by the WPCBAL score and PiPS-A. The NPV was the highest for the WPCBAL score, followed by the PiPS-B and PiPS-A. The AUROC was highest for the PiPS-B, followed by the WPCBAL score and PiPS-A. Only the PiPS-B exceeded 0.7, and all values had *p* < 0.001. There was no significant difference between the WPCBAL score, PiPS-A, and PiPS-B with respect to the AUROC.

### Feasibility rate for each prediction method

Table 4 shows the percentage of respondents for whom each prediction method was implemented, with the PPI having the highest percentage, followed by the PiPS-A, D-PaP, PaP, GPS, WPCBAL, and PiPS-B. The number and percentage of measurements performed for each test item in the WPCBAL score are shown in Supplementary Table S1A. In total, 96 patients (16.4% of all participants) were deficient in all six items of the WPCBAL score. This was the largest number of patients, followed by 51 patients (8.7%) who were deficient in only one item (Supplementary Table S1B). When we examined the breakdown of the 51 patients who were missing only one item, the least measured item among these 51 patients was LDH and was measured in only 13 patients (Supplementary Table S1C).

**Table 4.** Percentage of Each Prognostic Predictive Method Implemented

Methods	*N* (%)
PPI	577 (98.8%)
PiPS-A	475 (81.3%)
D-PaP	436 (74.7%)
PaP	436 (74.7%)
GPS	426 (72.9%)
WPCBAL score	264 (45.2%)
PiPS-B	203 (34.8%)

D-PaP, delirium-palliative prognostic score; GPS, Glasgow prognostic score; PaP, palliative prognosis score; PiPS, prognosis in palliative care study predictor models; PPI, Palliative Prognostic Index.

## Discussion

In this study, the utility of the WPCBAL score was directly compared with that of other prognostic methods. Our findings suggest that the predictive ability of the WPCBAL score is comparable to that of existing methods for predicting 2- and 3-week prognoses. As the six test items used in this study can be routinely measured outside Japan, we consider this method to be highly versatile.

Baba et al.^23^ conducted a multicenter prospective cohort study of 58 palliative care services in Japan to examine whether the PaP, D-PaP, PPI, PiPS-A, and PiPS-B were easier to use in general wards, palliative care wards, and homes. They reported that the PPI was best suited for routine clinical use in situations where patients did not want invasive procedures, such as blood draws, and that the PaP, D-PaP, and PiPS-B were appropriate if blood could be drawn or if a more accurate prediction was required. Therefore, depending on the context of its use, the WPCBAL score may be useful in predicting a 3-week prognosis when blood sampling is available.

In the 2-week prognosis projection, the WPCBAL score was more accurate than PiPS-A but less accurate than PiPS-B; however, the implementation rate of the WPCBAL score was higher than that of PiPS-B. Nevertheless, PiPS-B is impractical at a busy bedside. Therefore, to balance the accuracy and implementation rate, the WPCBAL score is considered a useful prognostic method for predicting a 2-week prognosis.

However, this study had some limitations. First, it was conducted exclusively at a single-center palliative care unit. Thus, we did not demonstrate the usefulness of the WPCBAL score in acute care wards or home settings. Future studies should examine the usefulness of the WPCBAL score in other settings such as multicenter acute care wards and home settings. For example, an ICU may be a good option for the WPCBAL score because significant amounts of blood are drawn and patients may be unable to provide information on their symptoms. Second, laboratory values could not be obtained from all hospitalized patients because this was an observational study. Third, this study comprised more excluded cases than a prospective intervention study, such as patients who required blood samples from inpatients who met the criteria. We chose this study design to assess its usefulness as a practical prediction method that reflects realistic situations in palliative care wards. However, a bias may have existed in the study population. For example, although we do not know if there was a difference between the included and excluded groups, the life expectancy could possibly be shorter in the included group than in the excluded group because blood is generally not drawn from patients in extremely poor health conditions. Therefore, it is necessary to evaluate the survival of an entire cohort in the future. Nevertheless, this study suggests that the WPCBAL score may be a new prognostic method for terminally ill patients with cancer by using only objective indicators that can predict prognosis at 2 or 3 weeks.

## Abbreviations Used

AIDS: acquired immune deficiency syndrome
ALB: albumin
ALP: alkaline phosphatase
ALT: alanine aminotransferase
AST: aspartate transaminase
AUROC: area under the receiver operating characteristic
BMI: body mass index
BUN: blood urea nitrogen
CRP: C-reactive protein
D-PaP: Delirium-Palliative Prognostic Score
ECOG PS: Eastern Cooperative Oncology Group Performance Status
GPS: Glasgow Prognostic Score
IQR: interquartile range
LDH: lactate dehydrogenase
NPV: negative predictive value
PLT: platelets
PPI: Palliative Prognostic Index
PPV: positive predictive value
PaP: Palliative Prognostic Score
PiPS: Prognosis in Palliative Care Study predictor models
WBC: white blood cells
WPCBAL score: combined score of six laboratory tests (white blood cells, platelets, C-reactive protein, blood urea nitrogen, aspartate transaminase, and lactate dehydrogenase
