# Supplementary material for: Is a Combination of Six Clinical Tests Useful as a Measure to Predict Short-Term Prognosis in Terminal Cancer Patients? A Prospective Observational Study in a Japanese Palliative Care Unit
Source: Palliat Med Rep. 2024 Oct 9;5(1):430–7. doi: 10.1089/pmr.2024.0026 (PMC11491580; doi:10.1089/pmr.2024.0026)
Supplement: Supplementary Table S1 [file pmr.2024.0026_supp_tables1.pdf]

Supplemental Table 1A. Number and percentage of measurements performed for each test item

	WBC	PLT	CRP	BUN	AST	LDH	ALB	Neutrophil count	ALT	ALP	Lymphocyte count	Lymphocyte percentage
n (%)	485 (83.0)	484 (82.9)	474 (81.2)	483 (82.7)	481 (82.4)	441 (75.5)	433 (74.1)	380 (65.1)	479 (82.0)	406 (69.5)	378 (64.7)	438 (75.0)

Supplemental Table 1B. Number and percentage of measurements performed for the six items of the WPCBAL score

Number of items measured	n (%)
6	426 (72.9)
5	51 (8.7)
4	7 (1.2)
3	2 (0.3)
2	2 (0.3)
1	0 (0.0)
0	96 (16.4)

Supplemental Table 1C. Number and percentage of items measured in cases where only one WPCBAL score item was not measured

	WBC	PLT	CRP	BUN	AST	LDH
n (%)	51 (100.0)	50 (98.0)	43 (84.3)	48 (94.1)	50 (98.0)	13 (25.5)
